# Supplementary figures and images for: Ecological filters shape arbuscular mycorrhizal fungal communities in the rhizosphere of secondary vegetation species in a temperate forest
Source: PLoS One. 2025 Jan 27;20(1):e0313948. doi: 10.1371/journal.pone.0313948 (PMC11771869; doi:10.1371/journal.pone.0313948)

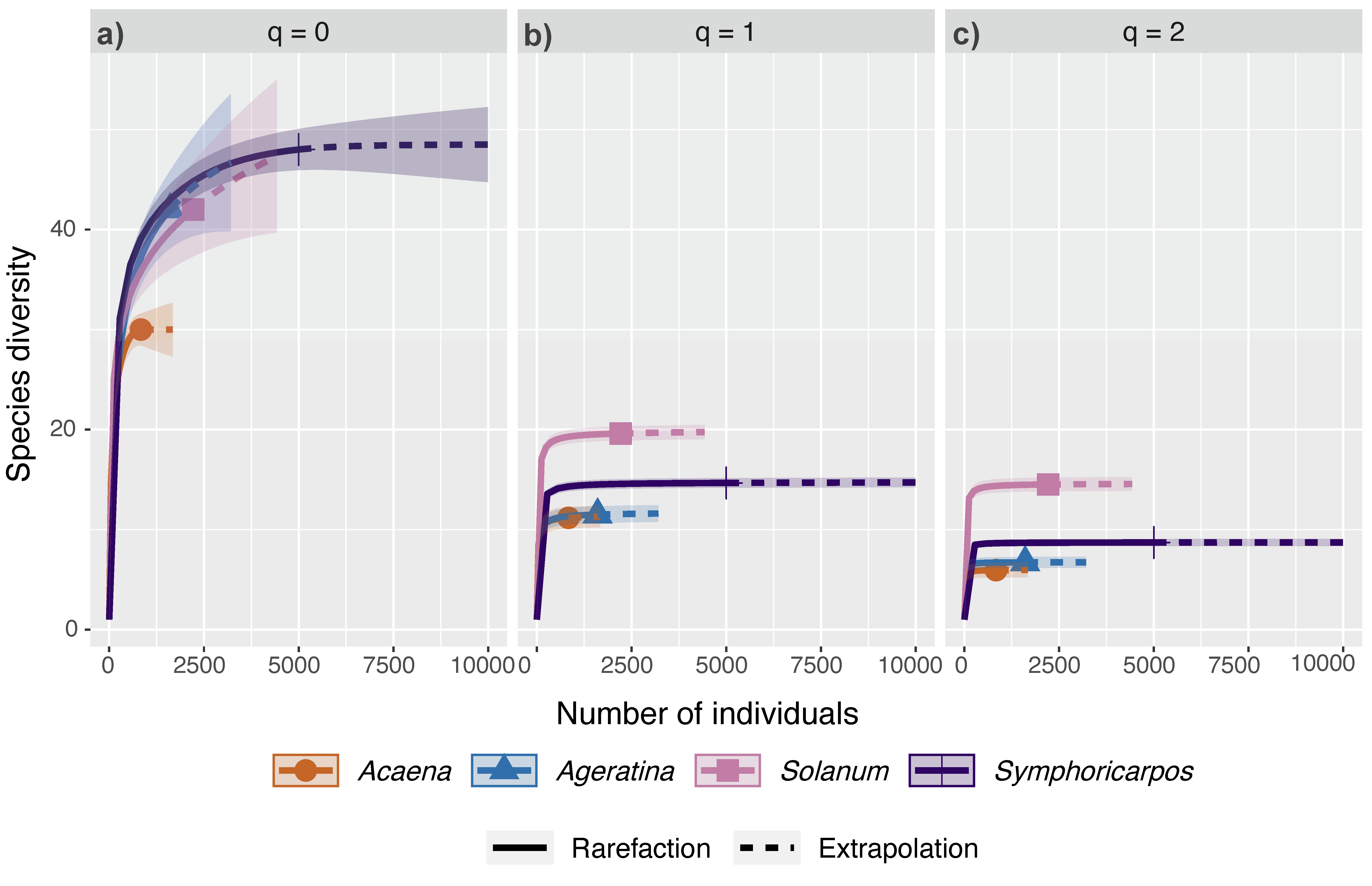

Supplement: S2 Fig — Acaena elongata (orange), Ageratina glabrata (blue), Solanum pubigerum (pink) and Syphoricarpos microphyllus (purple). a) Comparison of the effective species diversity (0D), b) typical species (1D) and c) dominant species (2D). The solid line indicates interpolation, the dotted line indicates extrapolation, and the shaded area represents the 95% confidence interval for each plant species. (TIFF) [file pone.0313948.s002.tiff]

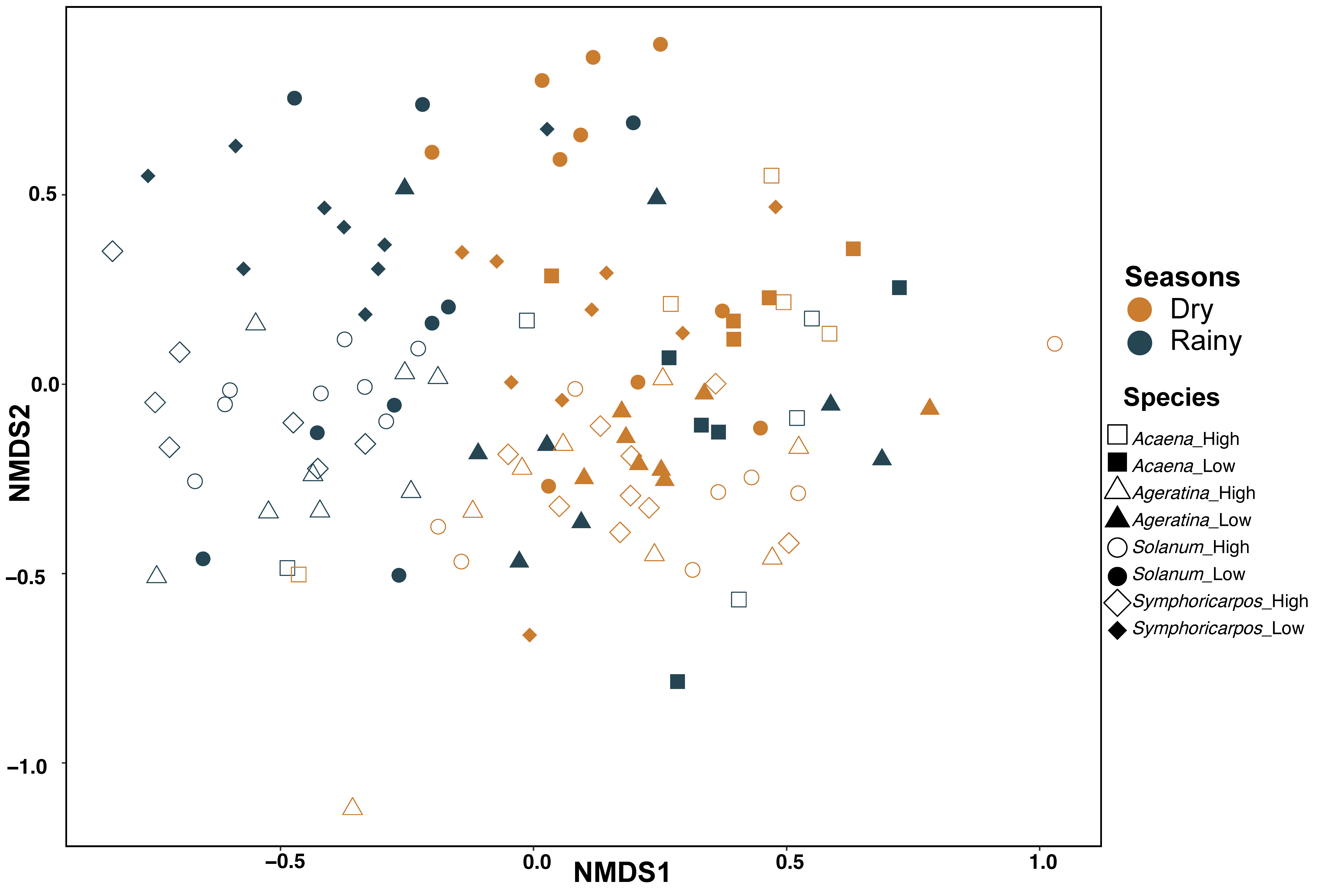

Supplement: S3 Fig — Non-metric multidimensional scaling showing the ordination of sampled individuals (dots) of each host plant species in the rainy and dry season and their location at high and low altitudes according to their AMF species composition, in the Abies religiosa forest of the Magdalena river basin, Mexico City, Mexico. The different shapes are related to the host plant species and altitude, and the different colors are related to the season (bluish green = rainy and orange = dry). (TIFF) [file pone.0313948.s003.tiff]

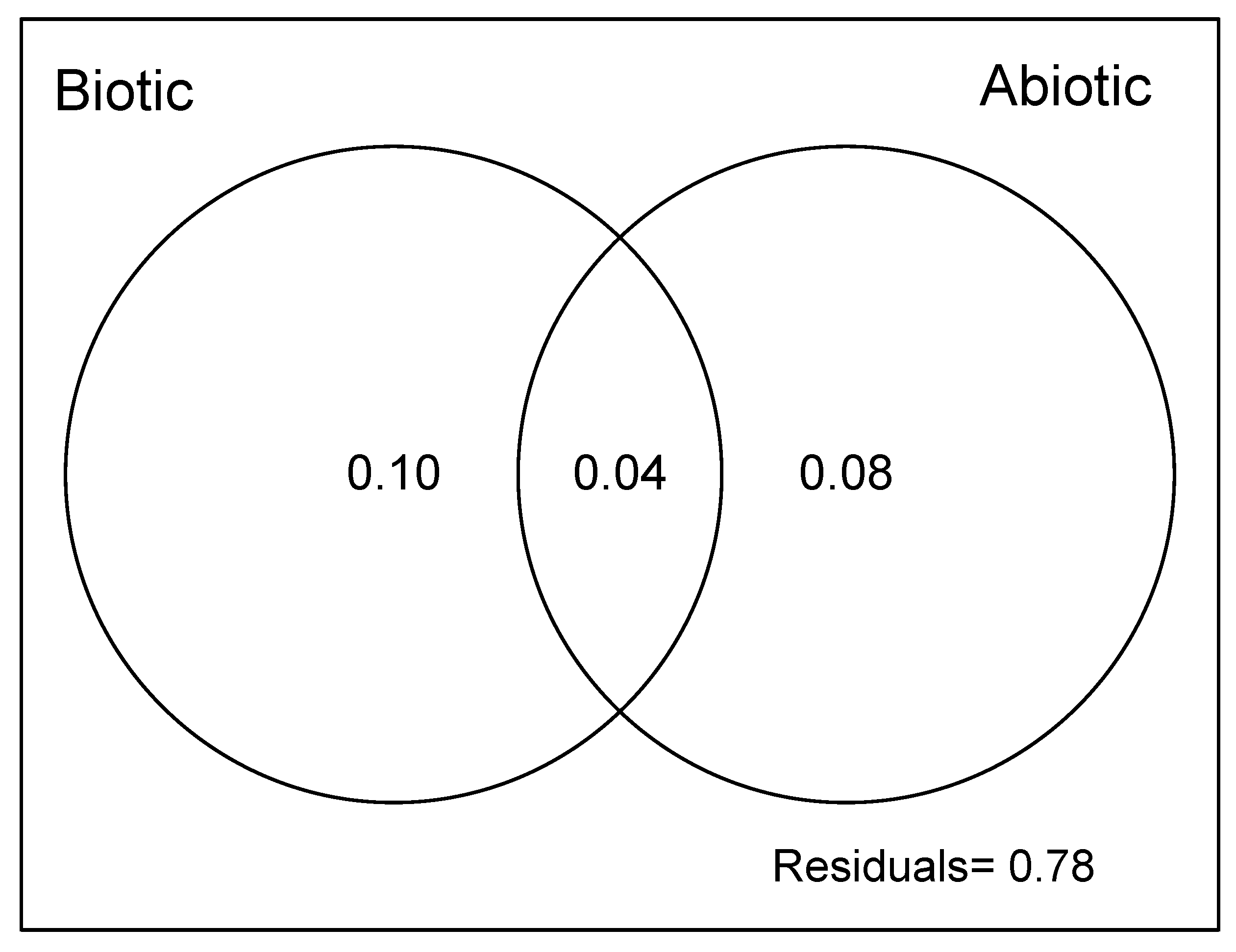

Supplement: S4 Fig — (TIFF) [file pone.0313948.s004.tiff]
