# Supplementary material for: Ecological filters shape arbuscular mycorrhizal fungal communities in the rhizosphere of secondary vegetation species in a temperate forest
Source: PLoS One. 2025 Jan 27;20(1):e0313948. doi: 10.1371/journal.pone.0313948 (PMC11771869; doi:10.1371/journal.pone.0313948)
Supplement: S1 Table — List of the arbuscular mycorrhizal fungi species registered in the rhizosphere soil of four plant species of secondary vegetation: Acaena elongata, Ageratina glabrata, Solanum cervantesii and Symphoricapos microphyllus during the rainy and the dry seasons in the Abies religiosa forest of the Magdalena river basin, Mexico City, Mexico. (DOCX) [file pone.0313948.s005.docx]

**S1 Table. Recording of arbuscular mycorrhizal fungi.** List of the arbuscular mycorrhizal fungi species registered in the rhizosphere soil of four plant species of secondary vegetation: *Acaena elongata, Ageratina glabrata*, *Solanum cervantesii* and *Symphoricapos microphyllus* during the rainy and the dry seasons in the *Abies religiosa* forest of the Magdalena river basin, Mexico City, Mexico.

| ***Host plant species*** | ***Acaena elongata*** | | ***Ageratina glabrata*** | | | ***Solanum pubigerum*** | | ***Symphoricarpos microphyllus*** | | | |
| --- | --- | --- | --- | --- | --- | --- | --- | --- | --- | --- | --- |
| **Season** | **Rainy** | **Dry** | **Rainy** | **Dry** | | **Rainy** | **Dry** | **Rainy** | | | **Dry** |
| **Glomales** | | | | | | | | | | | |
| **Claroideoglomeraceae** | | | | | | | | | | | |
| *Claroideoglomus claroideum* C. Walker & Schuessler | 0 | 3 | 4 | | 0 | 35 | 14 | 12 | 1 | | |
| *Claroideoglomus etunicatum* C.Walker & A.Schüßler | 8 | 8 | 15 | | 9 | 109 | 8 | 35 | 0 | | |
| *Claroideoglomus luteum* C.Walker & A. Schüßler | 0 | 0 | 0 | | 9 | 0 | 0 | 0 | 0 | | |
| **Glomeraceae** |  |  |  | |  |  |  |  |  | | |
| *Funneliformis caesaris* Oehl, G.A. Silva & Sieverd. | 0 | 0 | 0 | | 0 | 7 | 19 | 22 | 10 | | |
| *Funneliformis caledonius* C. Walker & Schuessler | 0 | 0 | 0 | | 0 | 0 | 0 | 14 | 0 | | |
| *Funneliformis coronatus* C. Walker & A. Schuessler | 0 | 0 | 2 | | 5 | 0 | 0 | 0 | 1 | | |
| *Funneliformis geosporus* C. Walker & Schuessler | 0 | 3 | 1 | | 33 | 8 | 14 | 2 | 29 | | |
| *Funneliformis mosseae* C. Walker & Schuessler | 20 | 12 | 7 | | 7 | 41 | 14 | 7 | 28 | | |
| *Funneliformis verruculosum* C. Walker & Schuessler | 0 | 0 | 2 | | 0 | 1 | 0 | 0 | 0 | | |
| *Glomus glomerulatum* Sieverd | 0 | 0 | 1 | | 0 | 0 | 0 | 2 | 0 | | |
| *Glomus heterosporum* G.S. Sm. & N.C. Schenck | 1 | 1 | 4 | | 1 | 0 | 43 | 0 | 20 | | |
| *Glomus spinuliferum* Sieverd. & Oehl | 0 | 0 | 0 | | 0 | 0 | 1 | 0 | 0 | | |
| *Glomus versiforme* S.M. Berch | 6 | 6 | 4 | | 1 | 14 | 1 | 0 | 0 | | |
| *Glomus* sp. 1 | 6 | 6 | 10 | | 7 | 0 | 34 | 0 | 35 | | |
| *Glomus* sp. 2 | 0 | 0 | 8 | | 0 | 18 | 0 | 26 | 0 | | |
| *Glomus Sp.3* | 0 | 0 | 0 | | 0 | 0 | 0 | 1 | 0 | | |
| *Rhizophagus clarus* C. Walker & A. Schüßler | 11 | 11 | 97 | | 36 | 60 | 28 | 38 | 45 | | |
| *Rhizophagus fasciculatus* C. Walker & Schuessler | 2 | 3 | 1 | | 2 | 3 | 0 | 5 | 2 | | |
| *Rhizophagus.* sp. 1 | 0 | 0 | 3 | | 0 | 0 | 0 | 0 | 0 | | |
| *Septoglomus fuscum* Błaszk. | 0 | 0 | 0 | | 0 | 0 | 0 | 447 | 0 | | |
| *Septoglomus viscosum* C. Walker, D. Redecker, S. Stille & A. Schussler | 0 | 0 | 3 | | 0 | 36 | 1 | 16 | 0 | | |
| *Sclerocystis* sp. 1 | 0 | 0 | 0 | | 4 | 0 | 0 | 0 | 0 | | |
| **Diversisporales** | | | | | | | |  | |  | |
| **Acaulosporaceae** | | | | | | | |  | |  | |
| *Acaulospora alpina* Oehl. Sýkorová & Sieverd | 1 | 1 | 1 | | 0 | 2 | 0 | 589 | 4 | | |
| *Acaulospora capsicula* Blaszk*.* | 0 | 0 | 0 | | 2 | 0 | 33 | 0 | 30 | | |
| *Acaulospora cavernata* Blaszk. | 3 | 3 | 1 | | 21 | 0 | 2 | 0 | 189 | | |
| *Acaulospora delicata* C. Walker, C.M. Pfeiff. & Bloss | 0 | 20 | 20 | | 1 | 0 | 316 | 2 | 10 | | |
| *Acaulospora elegans* Trappe & Gerd*.* | 0 | 0 | 0 | | 0 | 0 | 1 | 0 | 0 | | |
| *Acaulospora laevis* Gerd. & Trappe | 9 | 2 | 135 | | 63 | 109 | 50 | 99 | 125 | | |
| *Acaulospora longula* Spain & N.C. Schenck | 53 | 53 | 33 | | 440 | 0 | 231 | 0 | 324 | | |
| *Acaulospora mellea* Spain & N.C. Schenck | 32 | 268 | 31 | | 47 | 52 | 48 | 51 | 114 | | |
| *Acaulospora minuta* Oehl, Tchabi, Hountondji, Palez, I.C. Sánchez & G.A. Silva | 0 | 0 | 0 | | 0 | 0 | 0 | 0 | 2 | | |
| *Acaulospora morrowiae* Spain & N.C. Schenck | 20 | 26 | 67 | | 222 | 153 | 47 | 119 | 232 | | |
| *Acaulospora spinosa* C. Walker & Trappe | 1 | 1 | 2 | | 5 | 4 | 11 | 14 | 19 | | |
| *Acaulospora thomii* Blaszk | 0 | 0 | 0 | | 1 | 0 | 0 | 0 | 0 | | |
| A*caulospora* aff. *cavernata* Blaszk. | 0 | 2 | 20 | | 1 | 0 | 3 | 0 | 10 | | |
| *Acaulospora* aff*. ignota* Blaszk., Góralska, Chwat & Goto | 0 | 0 | 7 | | 3 | 0 | 3 | 11 | 4 | | |
| *Acaulospora* sp. 1 | 0 | 0 | 0 | | 0 | 0 | 0 | 3 | 0 | | |
| *Acaulospora* sp. 3 | 0 | 0 | 0 | | 0 | 0 | 0 | 2 | 0 | | |
| *Acaulospora* sp. 4 | 0 | 0 | 1 | | 0 | 0 | 0 | 2 | 0 | | |
| *Acaulospora* sp. 5 | 46 | 46 | 0 | | 0 | 63 | 0 | 26 | 0 | | |
| *Acaulospora* sp. 6 | 0 | 0 | 0 | | 0 | 0 | 5 | 0 | 0 | | |
| *Acaulospora* sp. 2 | 0 | 0 | 0 | | 0 | 0 | 45 | 0 | 3 | | |
| **Diversisporaceae** |  |  |  | |  |  |  |  |  | | |
| *Diversispora aurantia* C. Walker & A. Schuessler | 0 | 0 | 0 | | 5 | 0 | 1 | 0 | 20 | | |
| *Diversispora eburnea* C. Walker & Schuessler | 0 | 6 | 0 | | 0 | 0 | 21 | 0 | 0 | | |
| *Diversispora pustulata* Oehl, G.A. Silva & Sieverd | 9 | 9 | 15 | | 41 | 0 | 43 | 0 | 15 | | |
| *Diversispora trimurales* Walker & A. Schuessler | 8 | 8 | 11 | | 0 | 0 | 80 | 0 | 24 | | |
| **Gigasporaceae** |  |  |  | |  |  |  |  |  | | |
| *Cetraspora* pellucida Oehl, F.A. Souza & Sieverd. | 0 | 0 | 1 | | 0 | 0 | 0 | 0 | 0 | | |
| *Gigaspora decipiens* I.R. Hall & L.K. Abbott | 0 | 0 | 0 | | 0 | 1 | 0 | 0 | 0 | | |
| *Gigaspora gigantea* Gerd. & Trappe | 0 | 0 | 0 | | 0 | 1 | 0 | 0 | 1 | | |
| *Racocetra fulgida* Oehl, F.A. Souza & Sieverd | 0 | 0 | 0 | | 1 | 0 | 6 | 0 | 3 | | |
| *Scutellospora dipurpurescens* J.B. Morton & Koske | 1 | 1 | 1 | | 0 | 0 | 0 | 0 | 2 | | |
| *Sieverdingia tortuosa* Błaszk., Niezgoda & B.T. Goto | 1 | 1 | 0 | | 3 | 3 | 0 | 2 | 3 | | |
| **Pascisporaceae** |  |  |  | |  |  |  |  |  | | |
| *Pacispora chimonobambusae* C. Walker, Vestberg & Schuessler | 5 | 5 | 0 | | 0 | 0 | 0 | 1 | 0 | | |
| *Pacispora scintillans* Oehl & Sieverd. | 0 | 0 | 9 | | 25 | 10 | 0 | 15 | 36 | | |
| **Archeosporales** |  |  |  | |  |  |  |  |  | | |
| **Ambisporaceae** |  |  |  | |  |  |  |  |  | | |
| *Ambispora appendicula* C. Walker | 9 | 9 | 0 | | 3 | 0 | 0 | 0 | 117 | | |
| *Ambispora gerdemannii* C. Walker, Vestberg Schuessler | 3 | 3 | 25 | | 12 | 4 | 183 | 14 | 24 | | |
| *Ambispora reticulata* Oehl. & Sieverd. | 3 | 5 | 0 | | 0 | 0 | 0 | 0 | 4 | | |
| *Ambispora* sp. 1 | 1 | 1 | 10 | | 0 | 14 | 0 | 11 | 0 | | |
| **Archaeosporaceae** |  |  |  | |  |  |  |  |  | | |
| *Archaeospora trappei* J.B.Morton & D.Redecker | 0 | 11 | 0 | | 0 | 158 | 0 | 158 | 0 | | |
| **Paraglomales** |  |  |  | |  |  |  |  |  | | |
| **Paraglomeraceae** |  |  |  | |  |  |  |  |  | | |
| *Paraglomus albidum* Oehl, G.A.Silva & Sieverd | 23 | 23 | 5 | | 58 | 0 | 4 | 0 | 27 | | |
| **﻿Insertae sedis** |  |  |  | |  |  |  |  |  | | |
| *Entrophospora baltica* Blaszk., Madej & Tadych | 0 | 0 | 0 | | 2 | 0 | 0 | 0 | 0 | | |
| *Entrophospora infrequens* R.N. Ames & R.W. Schneid | 0 | 0 | 0 | | 0 | 0 | 1 | 1 | 3 | | |
