# Supplementary material for: Ecological filters shape arbuscular mycorrhizal fungal communities in the rhizosphere of secondary vegetation species in a temperate forest
Source: PLoS One. 2025 Jan 27;20(1):e0313948. doi: 10.1371/journal.pone.0313948 (PMC11771869; doi:10.1371/journal.pone.0313948)
Supplement: S2 Table — Analysis conducted for Canonical Correspondence Analysis (CCA) between edaphic-environmental factors and AMF community structure of four plant species in the Abies religiosa forest of the Magdalena river basin, Mexico City, Mexico. (DOCX) [file pone.0313948.s006.docx]

**S2 Table**. **Statistical summary of the Monte Carlo permutation test**. Analysis conducted for Canonical Correspondence Analysis (CCA) between edaphic-environmental factors and AMF community structure of four plant species in the *Abies religiosa* forest of the Magdalena river basin, Mexico City, Mexico.

| **Axis** | **Eigenvalue** | **Mean** | **Minimum** | **Maximum** | **P** |
| --- | --- | --- | --- | --- | --- |
| 1 | 0.557 | 0.374 | 0.248 | 0.679 | **0.016** |
| 2 | 0.309 | 0.254 | 0.114 | 0.424 |  |
| 3 | 0.188 | 0.180 | 0.083 | 0.302 |  |
