# Supplementary material for: Ecological filters shape arbuscular mycorrhizal fungal communities in the rhizosphere of secondary vegetation species in a temperate forest
Source: PLoS One. 2025 Jan 27;20(1):e0313948. doi: 10.1371/journal.pone.0313948 (PMC11771869; doi:10.1371/journal.pone.0313948)
Supplement: Attachment — Submitted filename: Response to Reviewers.docx [file pone.0313948.s007.docx]

Mexico City, Mexico, september 23th, 2024

**Emily Chenette**

**Editor-in-Chief**

PLOS ONE

Please find attached the new and corrected version of our manuscript entitled "Ecological filters shape arbuscular mycorrhizal fungal communities in the rhizosphere of secondary vegetation species in a temperate forest", authored by Yasmin Vázquez-Santos, Silvia Castillo-Argüero, Francisco Javier Espinosa-García, Noé Manuel-Montaño, Yuriana Martínez-Orea and Laura V. Hernández-Cuevas, which was accepted with changes in your Journal.

We attended all the suggestions and corrections to improve the manuscript quality.

Below we include the responses to the suggested changes (with some comments made by the reviewers and editor).

**Journal Requirements:**

1. Please ensure that your manuscript meets PLOS ONE's style requirements, including those for file naming. The PLOS ONE style templates can be found at

**Response: Following the PLOS ONE style templates, we made the style changes required by the journal.**

2. In your Methods section, please provide additional information regarding the permits you obtained for the work. Please ensure you have included the full name of the authority that approved the field site access and, if no permits were required, a brief statement explaining why.

**Response: We have included this information in the New Methods section.**

3. We note that your Data Availability Statement is currently as follows: All relevant data are within the manuscript and its Supporting Information files.

Please confirm at this time whether or not your submission contains all raw data required to replicate the results of your study. Authors must share the “minimal data set” for their submission. PLOS defines the minimal data set to consist of the data required to replicate all study findings reported in the article, as well as related metadata and methods (<https://journals.plos.org/plosone/s/data-availability#loc-minimal-data-set-definition>).

For example, authors should submit the following data:

- The values behind the means, standard deviations and other measures reported;
- The values used to build graphs

- The points extracted from images for analysis.

**Response: We have included in the manuscript and in the supplementary information all the information necessary to replicate our results.**

4. When completing the data availability statement of the submission form, you indicated that you will make your data available on acceptance. We strongly recommend all authors decide on a data sharing plan before acceptance, as the process can be lengthy and hold up publication timelines. Please note that, though access restrictions are acceptable now, your entire data will need to be made freely accessible if your manuscript is accepted for publication. This policy applies to all data except where public deposition would breach compliance with the protocol approved by your research ethics board. If you are unable to adhere to our open data policy, please kindly revise your statement to explain your reasoning and we will seek the editor's input on an exemption. Please be assured that, once you have provided your new statement, the assessment of your exemption will not hold up the peer review process.

**Response: We agree with all disclosure statements regarding data availability.**

5. We note that Figure 1 in your submission contain map/satellite images which may be copyrighted. All PLOS content is published under the Creative Commons Attribution License (CC BY 4.0), which means that the manuscript, images, and Supporting Information files will be freely available online, and any third party is permitted to access, download, copy, distribute, and use these materials in any way, even commercially, with proper attribution. For these reasons, we cannot publish previously copyrighted maps or satellite images created using proprietary data, such as Google software (Google Maps, Street View, and Earth). For more information, see our copyright guidelines:<http://journals.plos.org/plosone/s/licenses-and-copyright>.
 We require you to either (a) present written permission from the copyright holder to publish these figures specifically under the CC BY 4.0 license, or (b) remove the figures from your submission:

a. You may seek permission from the original copyright holder of Figure 1 to publish the content specifically under the CC BY 4.0 license.

We recommend that you contact the original copyright holder with the Content Permission Form (<http://journals.plos.org/plosone/s/file?id=7c09/content-permission-form.pdf>) and the following text:
“I request permission for the open-access journal PLOS ONE to publish XXX under the Creative Commons Attribution License (CCAL) CC BY 4.0 (<http://creativecommons.org/licenses/by/4.0/>). Please be aware that this license allows unrestricted use and distribution, even commercially, by third parties. Please reply and provide explicit written permission to publish XXX under a CC BY license and complete the attached form.”

**Response: We had asked for the permission to publish this figure to the author and included it as an attached material.**

6. Please include captions for your Supporting Information files at the end of your manuscript, and update any in-text citations to match accordingly. Please see our Supporting Information guidelines for more information: <http://journals.plos.org/plosone/s/supporting-information>.

Additional Editor Comments

**Response: We have verified that the manuscript meets all publication requirements.**

7. What parts do you consider original or relevant for the field?
The results and discussion are original and relevant to the fields of soil microbiology, and soil science. However, the authors could use more descriptive subsections by reducing the lenght of the results and discussion for being more punchy.

What specific improvements should the authors consider regarding the methodology? What further controls should be considered?

The authors could reduce the lenght of the results and discussion for being more punchy. Both sections are too long and hard to follow in the current version.

**Response: We have made great efforts to reduce the length of the results and discussion sections, and we have also emphasized our relevant findings on AMF species assemblages and the filters involved. We believe that our new version is easier to follow.**

8. Are the references appropriate? Yes, they are appropriate. However, the author could reduce the number of cited works.

**Response: We reduced the number of cited works.**

9. Please include any additional comments on the tables and figures and the quality of the data.
The tables and figures follow the authors' guidelines. However, all figures have low resolution and need to be improved. They are interesting, but hard to read and understand in the current version. The tables provide enough information and are well-presented.

**Response: We improved the resolution of all figures.**

Kind regards

Dr. Silvia Castillo-Argüero

Correspondence author
